# Weed Species Identification Using Hyperspectral Imaging and Machine Learning

**DOI:** 10.3390/plants15060916

**Published:** 2026-03-16

**Authors:** Rimma M. Ualiyeva, Mariya M. Kaverina, Anastasiya V. Osipova, Nurgul N. Iksat, Sayan B. Zhangazin

**Affiliations:** 1 Department of Biology and Ecology, Toraighyrov University, Pavlodar 140008, Kazakhstan; ualiyeva.r@gmail.com (R.M.U.); aanastasiyaaa@internet.ru (A.V.O.); 2 Department of Biotechnology and Microbiology, L.N. Gumilyov Eurasian National University, Astana 010000, Kazakhstan; nurguliksat@gmail.com

**Keywords:** hyperspectral imaging, weeds, spectral signatures, machine learning, automated identification, phytosanitary monitoring

## Abstract

Reliable identification of weed species is essential for effective and sustainable weed management. In this study, we explored the use of hyperspectral imaging to distinguish nine weed species based on their spectral signatures. Although the species showed similarities in their spectral curves due to comparable growing conditions, clear differences emerged related to morphological traits and pigment composition. We analysed the spectral data using five classification algorithms: Random Forest, Support Vector Machine, Artificial Neural Network, Maximum Entropy, and SIMCA. Model performance was assessed using per-class and overall accuracy. Random Forest outperformed the other methods, achieving 93.5% accuracy despite limited and imbalanced training data. This work contributes to the development of a spectral library for weed species and demonstrates the value of machine learning for species identification across different crops and environmental conditions. Expanding such spectral databases can enhance the speed and accuracy of weed monitoring, reduce herbicide reliance, and reduce environmental impact. The proposed approach shows strong potential for integration into precision agriculture and agroecological monitoring systems, supporting more efficient and environmentally responsible farmland management.

## 1. Introduction

Weed infestation remains one of the most significant threats to sustainable food production and the economic viability of agriculture. Weeds compete with crops for water, nutrients, and light, reducing both yield and product quality [1]. As a result, substantial crop losses are attributed to uncontrolled weed growth [2,3,4,5].

Traditional weed monitoring relies on visual scouting and manual sampling—methods that are labour-intensive, subjective, and poorly suited to large-scale or high-intensity farming. The growing scale of agricultural operations calls for more accurate, automated approaches to vegetation assessment. Hyperspectral imaging has emerged as a particularly promising solution [6].

When combined with machine learning, spectral signatures, RGB, and hyperspectral data enable reliable crop–weed discrimination and significantly enhance the accuracy of automated monitoring [7]. Computer vision and self-learning models [8,9] leverage differences in reflectance across red, near-infrared, and shortwave infrared regions to segment vegetation [10]. Wavelengths in the ranges of 700–750 nm and 900–970 nm are particularly sensitive to crop–weed differences [11]. The rise in UAV platforms has further expanded the practical reach of hyperspectral sensing, which now outperforms other optical technologies in weed identification accuracy [12]. Weed recognition studies using hyperspectral imaging have been conducted in rice [13,14,15], wheat [16], maize [6,17], soybean [18], tomato [19], and cabbage [20] fields, as well as in urban environments [21]. Applications include distinguishing weedy rice and barnyardgrass from cultivated rice [22], identifying *Centaurea maculosa* and *Gypsophila paniculata* [23], classifying invasive species in alpine meadows [24], separating grasses from broadleaf weeds [25,26], and differentiating *Cyperus esculentus* from morphologically similar species [27]. These classifications are possible due to differences in leaf spectral properties driven by pigment composition, water content, physiological status [28], and growth stage [29]. Hyperspectral imaging has also been used to track crop–weed competition for water [30], detect early physiological stress in crops [31], assess weed impact on yield [32], and estimate infestation levels [33].

A range of classification algorithms has been employed, including Spectral Angle Mapper, Mahalanobis distance [34], SVM, ANN [35], Random Forest [6], CNN [7], partial least squares-discriminant analysis (PLS-DA), and Multilayer Perceptron [25], with reported accuracies between 90 and 99% [11,20,35,36]. Feature selection and classification have also supported selective robotic weeding [37]. To address challenges such as high dimensionality, computational load, mixed pixels, illumination variability, and sensor cost and weight, researchers are exploring dimensionality reduction (band selection, spectral indices), spectral decomposition algorithms (unmixing), and HSI integration with other technologies (LiDAR or multispectral systems) [12,38,39]. Preprocessing techniques (denoising, normalisation), band selection, and optimised neural architectures improve classification robustness under variable field conditions [31,38]. HSI-based systems are increasingly viewed as viable tools for precision agriculture and site-specific herbicide application [40]. These systems reduce chemical inputs, lower environmental impact [1,12], minimise crop damage, and achieve weed removal rates above 90% [41]. Public datasets are now emerging, including ground-based hyperspectral images of crops (canola, soybean, sugar beet) and weeds (kochia, common waterhemp, redroot pigweed, and common ragweed) [42], as well as controlled-condition datasets of *Amaranthus retroflexus*, *Solanum nigrum*, *Setaria adhaerens* [43].

Over the past decade, the number of studies demonstrating the potential of HSI for weed species differentiation and infestation assessment has grown considerably [1,25,44,45]. However, despite these advances, research focused on applying hyperspectral imaging to weed identification and mapping in Central Asian wheat systems remains scarce. Most existing work targets rice, maize, or soybean crops, while systematic investigations into the spectral properties of weeds in steppe and semi-arid environments—characteristic of Kazakhstan, Uzbekistan, and neighbouring regions—remain extremely limited. Given that weeds in wheat fields represent one of the most persistent and economically damaging challenges in Central Asian agriculture, causing substantial yield losses, grain quality deterioration, and increased chemical control costs, targeted research on hyperspectral monitoring of weeds in Central Asian wheat agrocenoses is urgently needed.

This study aims to identify spectral patterns in nine weed species and develop a classification model for their reliable identification.

## 2. Results

The figures presented below show hyperspectral images of weed species visualised using false-colour mapping with colour intensity representing reflectance (%): cool colours (blue, cyan) indicate low-reflectance areas, while warm colours (red, orange) represent high-reflectance zones. The Raw Spectrum plots display spectral signatures for different plant parts as reflectance coefficients across wavelengths. For each sample, specific zones were selected under PCA mode based on reflectance intensity. The first Raw Spectrum plot shows data for colour-coded zones; the second plot corresponds to manually selected plant structures (e.g., flower, leaf, stem, root). In all figures, a colour scale links the reflectance spectra to the corresponding false-colour hyperspectral image, while a legend beneath the second Raw Spectrum plot identifies each weed species. The Variance Scatter plots show the distribution of spectral data after hypercube dimensionality reduction, with each point representing a single pixel projected onto principal component coordinates.

**Figure 1:**
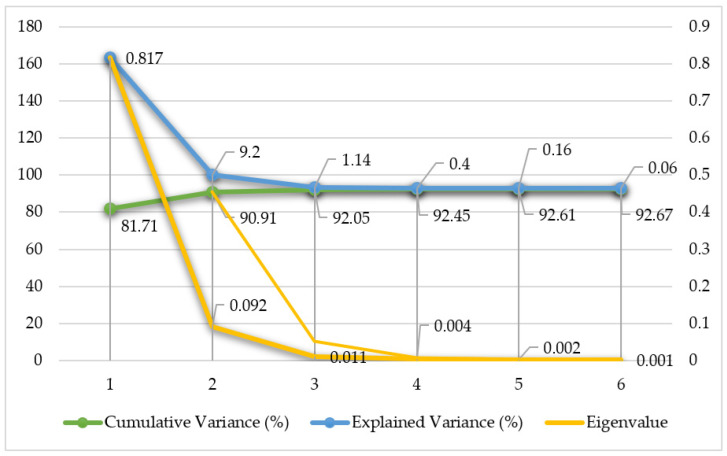
Scree plot of explained variance.

Variance analysis focused on the first two principal components, as they capture the largest share of total spectral variance. Detailed explained variance values are presented in Table 1 and Table 2 and Figure 1.

The first two principal components together account for approximately 90.9% of cumulative variance. This indicates that over 90% of the total spectral variability across all sampled weed species can be described using only these two components. The eigenvalues for PC1 and PC2 (0.817 and 0.092, respectively) confirm their high information content relative to subsequent components. Given that the first two principal components explain the overwhelming majority of data variance, a two-dimensional space was adopted for visualising spectral data distribution and conducting subsequent analysis of major spectral differences.

### 2.1. Spectral Characteristics

#### 2.1.1. *Convolvulus arvensis*

**Figure 2:**
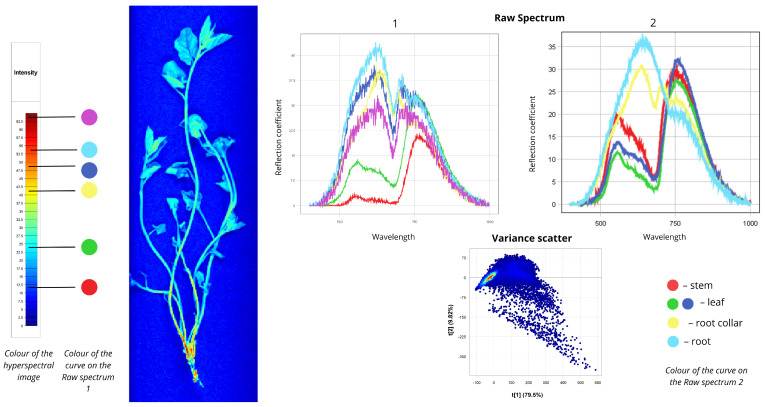
Spectral signatures of the *Convolvulus arvensis*.

Figure 2 presents the spectral data for field bindweed (*Convolvulus arvensis*). In the first Raw Spectrum plot, the red curve represents spectral signatures of regions highlighted in blue in the hyperspectral image, representing leaf veins and shaded regions. The first peak of this curve occurs at 5% reflectance, and the second at 22.5%. The green curve represents most of the plant body—stems and leaves—shown in cyan in the hyperspectral image, with reflectance values of 13% at the first peak and 32% at the second. The yellow, blue, cyan, and purple curves correspond to the basal parts of the plant, with reflectance values ranging from 30% to 45%. In the second Raw Spectrum plot, the root exhibits the highest intensity: the first peak reaches 35% reflectance in the 550–750 nm range, followed by a second peak of 20% in the 750–780 nm range. Moderate intensity is observed for the root collar and leaf spectra. The root collar curve shows 30% reflectance in the 550–700 nm range and 25% in the 700–750 nm range. By contrast, leaf and stem curves display distinctly different shapes: a less pronounced peak in the 550–700 nm region and a more prominent peak in the 750–780 nm region corresponding to the red edge. Leaf reflectance measures 10–12% at the first peak and 26–31% at the second, while stem reflectance reaches 20% at the first peak and 30% at the second. The Variance Scatter plot shows the first component (t [1] = 79.5%) capturing most of the spectral variation and differences between spectra, while the second component (t [2] = 9.82%) accounts for additional variability. Most points form clusters, indicating similar spectral signatures among many pixels. Outliers represent root zones with distinct spectral signatures.

#### 2.1.2. *Erigeron canadensis*

**Figure 3:**
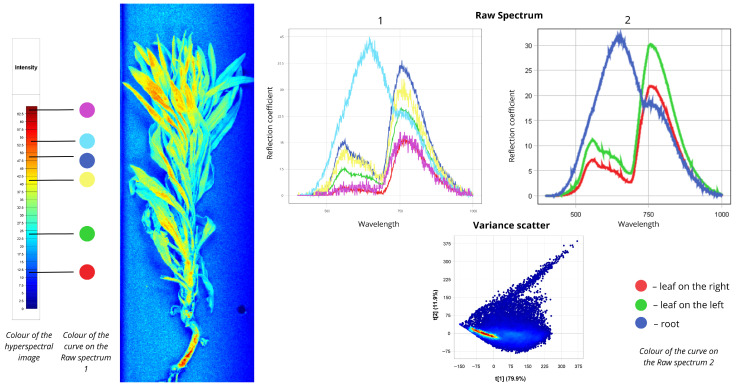
Spectral signatures of the *Erigeron canadensis*.

Figure 3 shows the spectral data for Canadian horseweed (*Erigeron canadensis*). In the first Raw Spectrum plot, the red curve corresponds to leaf areas coloured blue in the false-colour image. The first peak shows 5% reflectance in the 500–700 nm range, the second reaches 15% between 700 nm and 780 nm. Green and yellow curves represent leaf zones shown in cyan, light green, and yellow. The green curve’s first peak shows 7.5% reflectance in the 500–700 nm range, with a second peak at 22.5% in the 700–780 nm range. The yellow curve exhibits 13% reflectance at the first peak (500–700 nm) and 30% at the second (700–780 nm). The blue curve corresponds to leaf areas coloured orange, with 15% reflectance at the first peak and comparable second-peak values within 700–780 nm. The cyan curve differs markedly in shape and corresponds to root tissue. Its first peak reaches 45% reflectance in the 600–750 nm range, while the second peaks at 22.5% in the 750–780 nm range. In the second Raw Spectrum plot, the root again shows the highest intensity: 30% reflectance in the 550–750 nm region and 19% in the 750–780 nm region. Sun-exposed leaves exhibit moderate intensity (11% in the 550–700 nm range, 30% in the 700–780 nm range). Shaded leaves show the lowest reflectance: 7% at the first peak (550–700 nm) and 22% at the second (700–780 nm). The Variance Scatter plot indicates that the first component (t [1] = 79.9%) captures most spectral information, while the second component (t [2] = 11.9%) accounts for additional variation.

#### 2.1.3. *Erysimum cheiranthoides*

**Figure 4:**
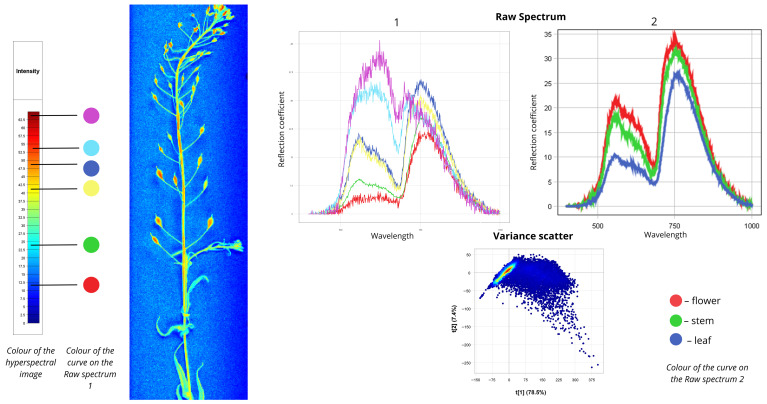
Spectral signatures of the *Erysimum cheiranthoides*.

Figure 4 presents spectral data for wormseed mustard (*Erysimum cheiranthoides*). In the first Raw Spectrum plot, all curves exhibit peaks in the 500–700 nm range and again near the red edge (700–780 nm). The red curve corresponds to blue areas in the hyperspectral image, with reflectance values of 5% in the first range and 22.5% in the second. The green spectrum describes cyan-coloured leaf zones: 7.5% reflectance in the first range and 25% in the second. Blue and yellow curves represent stem zones with yellow-orange false colours; both show 20% reflectance in the first range, with the yellow curve reaching 30% in the second range and the blue curve reaching 35%. Cyan and purple spectra correspond to flower zones and exhibit distinct shapes with higher peaks in the visible range: cyan shows 35% reflectance in the first range and 30% in the second; purple shows 40% in the first range and 30% in the second. In the second Raw Spectrum plot, all curves share similar shapes. Flowers display the highest intensity (from 20% in the 500–700 nm range to 35% in the 700–780 nm range). Stems show moderate intensity (18% in the 500–700 nm range to 30% in the 700–780 nm range). Leaves exhibit the lowest reflectance (from 10% in the 500–700 nm range to approximately 25% in the 700–780 nm range). The Variance Scatter plot shows the first component (t [1]) accounting for approximately 78.5% of total variance, while the second component (t [2]) explains only 7.4%.

#### 2.1.4. *Sonchus arvensis*

**Figure 5:**
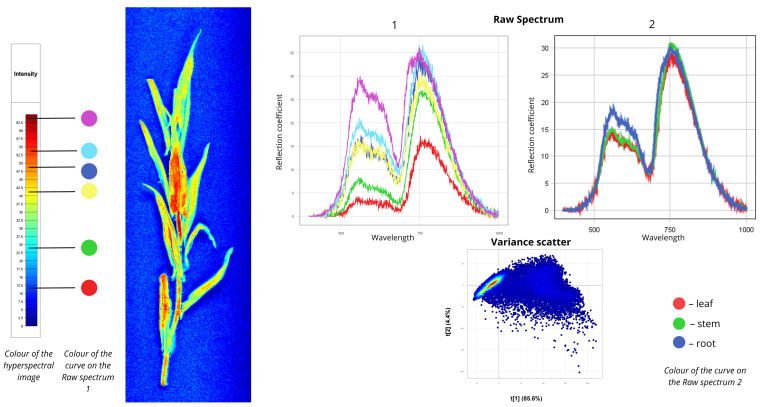
Spectral signatures of the *Sonchus arvensis*.

Figure 5 presents spectral data for perennial sowthistle (*Sonchus arvensis*). In the first Raw Spectrum plot, the red curve describes isolated blue zones in the hyperspectral image, with reflectance values of approximately 3% in the 500–700 nm range and 15% in the 700–780 nm range. The green curve represents trichomes coloured cyan, with peak reflectance ranging from 7% (500–700 nm) to 25% (700–780 nm). Leaves are shown in light green and yellow, corresponding to the yellow curve: 15% reflectance at the first peak (500–700 nm) and 30% at the second (700–780 nm). Stems and certain leaf areas coloured red and orange are represented primarily by blue, cyan, and purple curves. The blue spectrum shows peaks at 15% (500–700 nm) and 30% (700–780 nm). The cyan curve exhibits 20% reflectance at the first peak (500–700 nm) and 35% at the second (700–780 nm). The purple spectrum demonstrates peaks at 30% (500–700 nm), increasing to 35% near the red edge.

In the second Raw Spectrum plot, all curves share an identical shape, transitioning from a lower to a more pronounced peak, with similar intensity patterns: the root shows approximately 19% reflectance in the 500–700 nm range and 30% in the 700–780 nm range; the stem corresponds to 15% and 30%; leaves show 15% and 28%, respectively. The Variance Scatter plot reveals that the first component (t [1]) accounts for 85.6% of total variance, while the second component (t [2]) explains only 4.4%.

#### 2.1.5. *Capsella bursa-pastoris*

**Figure 6:**
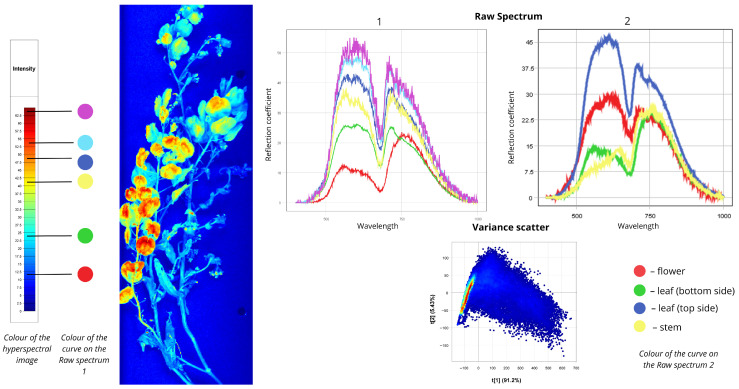
Spectral signatures of the *Capsella bursa-pastoris*.

Figure 6 shows spectral data for shepherd’s purse (*Capsella bursa-pastoris*). All spectra in the first Raw Spectrum plot exhibit similar shapes, with the first peak in the visible range (500–700 nm) and the second near the red edge (700–780 nm). The red curve corresponds to shaded branches and leaves in the hyperspectral image (10–22% reflectance). The green curve represents leaf zones coloured cyan (25%). The yellow curve corresponds to sun-exposed yellow leaf areas (35%). The cyan, blue, and purple spectra reflect red-coloured light-exposed leaf zones (40–50%). In the second Raw Spectrum plot, sun-exposed leaves show the highest intensity (45% and 37.5% in the respective ranges). Flowers exhibit moderate intensity (30% in the 500–700 nm range, 22.5% in the 700–780 nm range), with both tissue types sharing similar curve shapes. Lower reflectance intensities are observed for stems and abaxial leaf surfaces (ranging from 15% to 22.5%). The Variance Scatter plot shows the first component (t [1]) accounting for 91.2% of total variance, while the second component (t [2]) contributes only 5.43%.

#### 2.1.6. *Artemisia vulgaris*

**Figure 7:**
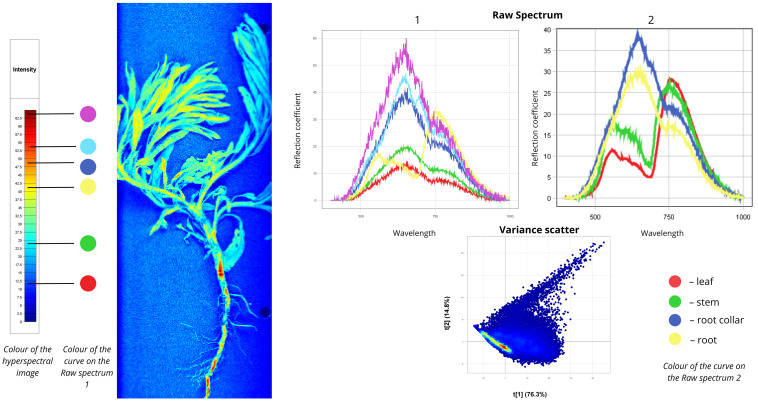
Spectral signatures of the *Artemisia vulgaris*.

Figure 7 presents spectral data for common mugwort (*Artemisia vulgaris*). In the first Raw Spectrum plot, the red curve reflects shaded blue leaf zones (10–12%). The green spectrum describes most leaf areas (10–20%). The yellow curve corresponds to sun-exposed yellow leaf zones (18–30%). Blue, cyan, and purple spectra clearly show reflectance from the basal (20–45%) and root portions (30–55%). The first major peaks occur in the 550–750 nm range, while second peaks of lower intensity appear mainly in the 750–780 nm range. The yellow curve exhibits a distinct spectral shape, with the lowest peak in the 500–700 nm region and the highest near the red edge (700–780 nm). In the second Raw Spectrum plot, basal and root portions show high intensity compared to leaves and stems. The basal part exhibits 40% reflectance in the 550–750 nm range and 15% near the red edge (750–780 nm). The root shows 30% reflectance in the green-red spectral zone (550–750 nm) and 15% within the red edge, matching the basal portion. Both lower plant parts share similar curve shapes. Stems and leaves, by contrast, display different patterns: the stem (green spectrum) shows 15% reflectance in the VIS region and 25% within the red edge. The red curve represents leaf spectra with two peaks: 10% in the green-red visible portion and 28% in the transitional region between visible and near-infrared ranges. The Variance Scatter plot indicates that the first component (t [1]) accounts for 76.3% of total variance, while the second component (t [2]) explains approximately one-seventh of all data (14.8%).

#### 2.1.7. *Ambrosia artemisiifolia*

**Figure 8:**
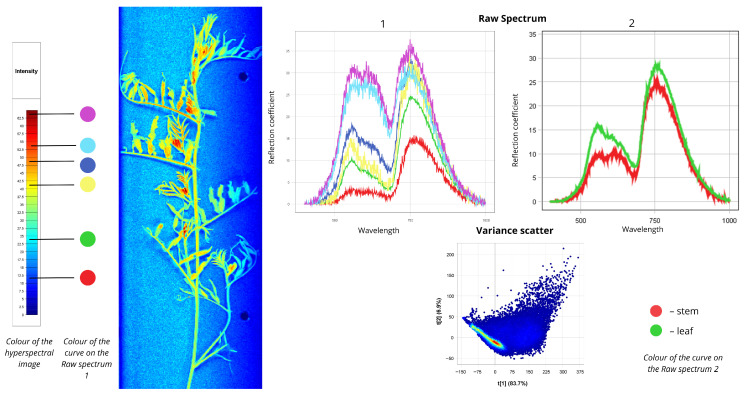
Spectral signatures of the *Ambrosia artemisiifolia*.

Figure 8 presents spectral data for common ragweed (*Ambrosia artemisiifolia*). In the first Raw Spectrum plot, all curves share similar shapes, with the first peak in the longwave visible region (500–700 nm) and the second in the VIS-NIR transition zone (700–780 nm). The red curve represents shaded leaves: reflectance is approximately 3% in the first range, increasing to nearly 15% in the second. Shaded leaves coloured cyan are described by the green curve, with reflectance values of 10% in the first range and 25% in the second. Most leaf zones are captured by the yellow spectrum, showing maximum reflectance of 15% and 33%. Isolated orange-coloured leaf areas appear as the blue curve (18–32%), red false-coloured leaves as the cyan curve (28–30%), and burgundy-coloured zones receiving direct light as the purple curve (30–35%).

In the second Raw Spectrum plot, reflectance intensity and spectral shapes are nearly identical across tissues. In the 500–700 nm range, leaves show 15% reflectance while stems show 10%. Within the red edge region (700–780 nm), leaves reach 29% reflectance, while stems exhibit slightly lower intensity at 25%. The Variance Scatter plot shows the first component (t [1]) accounting for 83.7% of total variance, with the second component (t [2]) explaining 6.9%.

#### 2.1.8. *Amaranthus retroflexus*

**Figure 9:**
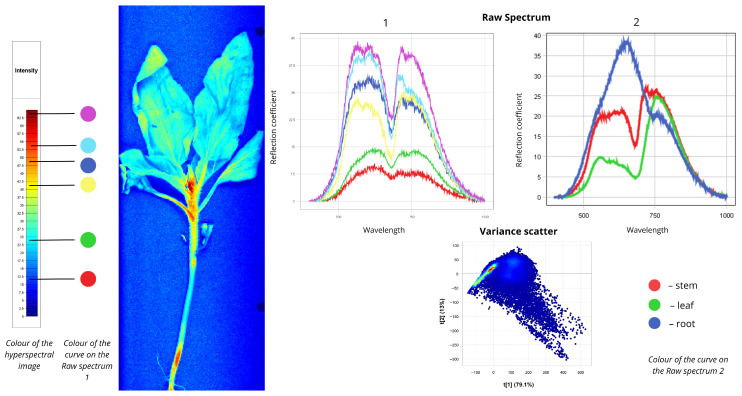
Spectral signatures of the *Amaranthus retroflexus*.

Figure 9 shows the dimensionality-reduced hyperspectral image for redroot pigweed (*Amaranthus retroflexus*). In the first Raw Spectrum plot, the red curve corresponds to blue-coloured leaf veins and shaded areas in the hyperspectral image. The first peak occurs at 8% reflectance, the second at 7.5%. The green spectrum represents most of the plant, primarily leaf zones coloured cyan, with reflectance of 14% at both peaks. The yellow curve describes stem areas and adjacent leaf veins, with reflectance values of 28% and 30%. Blue, cyan, and purple curves reflect the upper branched stem portion and root zone, with reflectance ranging from 28% to 43%. The first spectral peaks occur in the longwave visible region (500–700 nm), and the second in the red edge and near-infrared transition zone (700–780 nm). In the second Raw Spectrum plot, the root shows the highest intensity: 40% reflectance in the 550–750 nm range and 20% in the 750–780 nm range. Stems and leaves exhibit lower reflectance values: 20% and 10%, respectively, in the 550–700 nm range; both show approximately 25% reflectance in the VIS-NIR transition zone. The Variance Scatter plot indicates that the first component (t [1]) represents 79.1% of total variance, while the second component (t [2]) accounts for 13%.

#### 2.1.9. *Chenopodium album*

**Figure 10:**
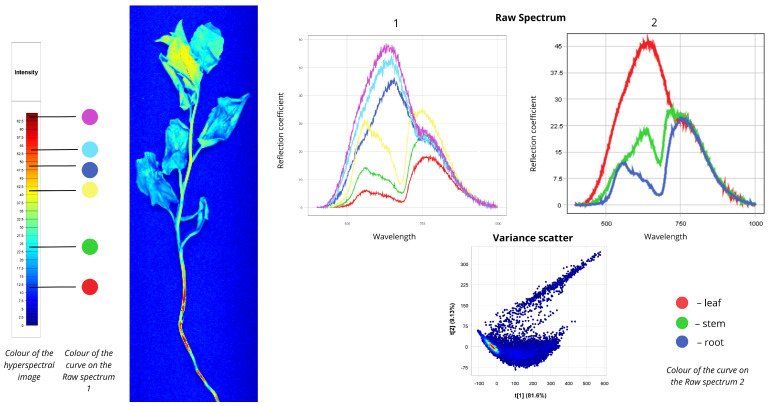
Spectral signatures of the *Chenopodium album*.

Figure 10 presents spectral information for common lambsquarters (Ch *Chenopodium album*). In the first Raw Spectrum plot, the red, green, and yellow curves show their first peaks in the VIS range (500–700 nm) and second peaks in the NIR region (700–780 nm). The blue, cyan, and purple curves exhibit first peaks in the VIS-red edge range (500–750 nm) and second peaks in the 750–780 nm NIR region. The red curve describes blue-coloured leaf veins (7–19% reflectance). The green curve represents cyan false-coloured leaves (15–25%). The yellow curve reflects the upper leaf and stem zone (30–35%). The root appears in orange and red false colours, described by blue (45% and 25%), cyan (50% and 25%), and purple (55% and 25%) curves. In the second Raw Spectrum plot, leaves show a first peak at 45% reflectance in the 500–750 nm range and a second peak at 22.5% in the 750–780 nm range. Stems exhibit a first peak at 22% in the 550–700 nm range and a second at 25% in the 700–780 nm range. Roots show a first peak not exceeding 10% in the 500–700 nm range, with a second peak at approximately 22.5% in the NIR region. The Variance Scatter plot reveals that the first component (t [1]) accounts for 81.6% of total variance, while the second component (t [2]) explains 9.13%.

#### 2.1.10. General Spectral Characteristics

**Figure 11:**
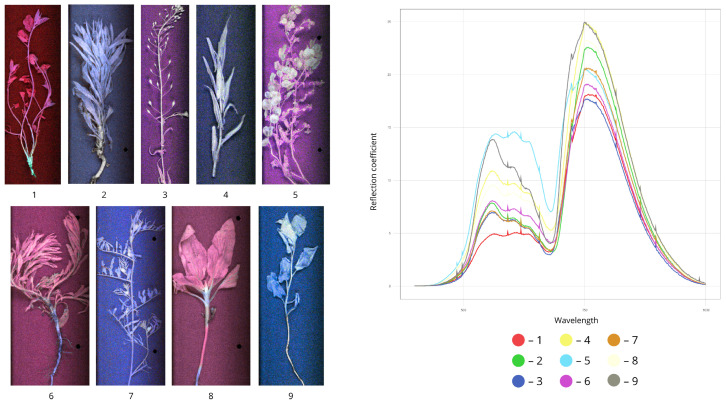
Spectral characteristics of weeds (pseudo-RGB image) (1—*Convolvulus arvensis*, 2—*Erigeron canadensis*, 3—*Erysimum cheiranthoides*, 4—*Sonchus arvensis*, 5—*Capsella bursa-pastoris*, 6—*Artemisia vulgaris*, 7—*Ambrosia artemisiifolia*, 8—*Amaranthus retroflexus*, 9—*Chenopodium album*).

Figure 11 presents an overall Raw Spectrum plot for all weed species sampled. Notably, all curves exhibit similar spectral shapes.

The analysed spectral response range covered two regions: the visible spectrum (500–700 nm) and the near-infrared region (700–780 nm). All species exhibited higher reflectance values in the NIR range compared to the VIS region, consistent with the typical increase in green biomass reflectance across the red edge.

In the visible range, the lowest reflectance value was observed for *Convolvulus arvensis* (5%), while the highest was recorded for *Capsella bursa-pastoris* and *Chenopodium album* (14%). Other weed species showed intermediate reflectance values in this region: *Erigeron canadensis* (8%), *Erysimum cheiranthoides* (6%), *Sonchus arvensis* (11%), *Artemisia vulgaris* (8%), *Ambrosia artemisiifolia* (6%), and *Amaranthus retroflexus* (9%).

In the near-infrared region, minimum reflectance was observed for *Erysimum cheiranthoides* (16%), while maximum reflectance was recorded for *Amaranthus retroflexus* (26%). Intermediate NIR reflectance values were found for: *Convolvulus arvensis* (17%), *Erigeron canadensis* (23%), *Sonchus arvensis* (25%), *Capsella bursa-pastoris* (21%), *Artemisia vulgaris* (19%), *Ambrosia artemisiifolia* (21%), and *Chenopodium album* (25%).

Regardless of weed species, all spectra showed a gradual increase in reflectance when transitioning from the VIS to the NIR region. These data served as the basis for quantitative comparison of spectral characteristics across different weed species.

### 2.2. Statistical Analysis

Based on the reflectance and wavelength data obtained during hyperspectral imaging, key statistical variability parameters were calculated. Table 3 presents the main reflectance metrics for each species. Mean reflectance values (μ) for generalised spectral curves ranged from 18–20.5% to 24.5–24.8%, with *Ambrosia artemisiifolia*, *Erysimum cheiranthoides*, *Capsella bursa-pastoris*, and *Chenopodium album* occupying the upper end of this range. These values serve as identifiers of each species’ optical properties. For the remaining weed species, both mean reflectance and median values fell within the 20.5–22.6% range.

Standard deviation (σ), indicating moderate intraspecific variability in spectral response, ranged from 2.1 to 2.8. The highest dispersion in reflectance was observed for *Erigeron canadensis*, while the lowest was recorded for *Convolvulus arvensis*. The coefficient of variation, calculated to assess relative variability, revealed relatively stable spectral patterns within each species, ranging from 8.7% (*Capsella bursa-pastoris*) to 13.6% (*Ambrosia artemisiifolia*). The close alignment between median values and mean reflectance (μ) confirms the representativeness of the mean values, particularly for describing weed spectral characteristics, and indicates the absence of pronounced asymmetry in reflectance distribution. The rate of change in reflectance and delta reflectance (ΔR) exhibited similar trends across species, ranging from 10.9% (R) and 16.8% (ΔR) for *Sonchus arvensis* to 14.8% (R) and 22.3% (ΔR) for *Erigeron canadensis*. These values reflect species-specific differences in spectral response patterns.

**Figure 12:**
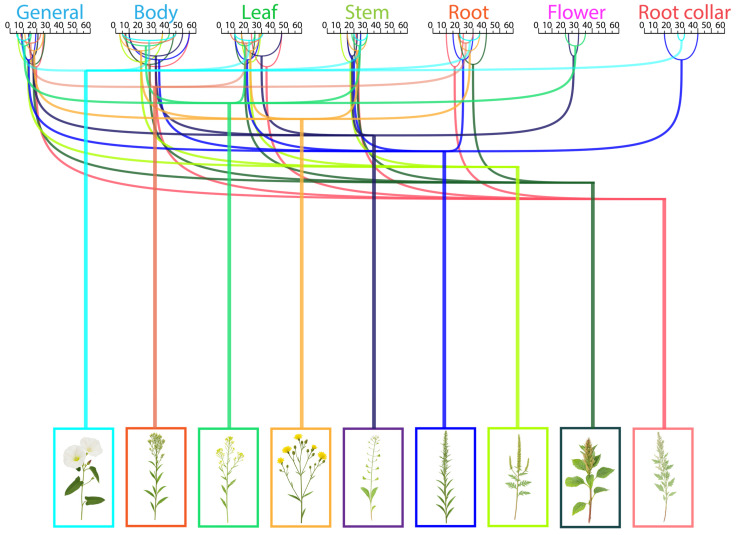
Spectral response patterns based on coefficients of reflectance.

Analysis of reflectance ranges across different morphological parts of each plant revealed that maximum values were predominantly associated with leaves and above-ground biomass. In contrast, root and basal structures displayed more constrained reflectance ranges, indicating that foliar organs contribute most significantly to the integrated spectral signatures of weed species (Figure 12).

Examination of spectral response within the operational wavelength range showed that most morphological parts—and whole plants overall—exhibited spectral activity primarily between 500 and 780 nm (Table 4). This range encompasses the visible and near-infrared regions. For certain morphological structures, particularly stems, roots, and basal zones, the effective range was narrower, starting at approximately 550 nm. This finding indicates a more restricted spectral channel response in these plant parts.

Spectral bandwidth (SB) showed the greatest variability among the wavelength-based metrics across species. Maximum SB values reached 280 nm for *Erysimum cheiranthoides*, *Sonchus arvensis*, *Capsella bursa-pastoris*, *Ambrosia artemisiifolia*, indicating broader spectral ranges over which reflectance characteristics are distributed. More concentrated spectral responses were observed for *Amaranthus retroflexus* (SB = 238 nm) and *Convolvulus arvensis* (SB = 246.7 nm).

**Figure 13:**
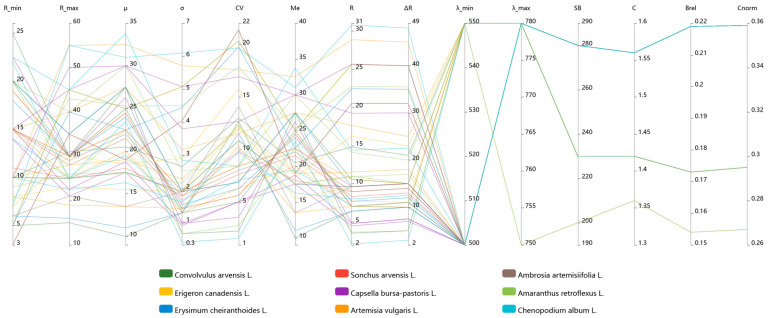
Statistical indicators of spectral variability in weed species.

The contrast ratio exhibited relatively limited variation, suggesting moderate spectral differentiation between species in terms of brightness: values ranged from 1.453 (*Amaranthus retroflexus*) to 1.560 (*Erysimum cheiranthoides*, *Sonchus arvensis*, *Capsella bursa-pastoris*, *Ambrosia artemisiifolia*). Relative bandwidth values fell within 0.184–0.219, indicating comparability of spectral metrics across weed species despite individual biological differences. Similar patterns emerged for the normalised contrast coefficient (C_norm_ = 0.309–0.359), reflecting broadly similar differences between spectral troughs and peaks within the studied wavelength range. A consistent relationship was observed between normalised contrast and spectral bandwidth: the highest values of the former correspond to the maximum value of the latter (*Erysimum cheiranthoides*, *Sonchus arvensis*, *Capsella bursa-pastoris*, *Ambrosia artemisiifolia*) (Figure 13).

In summary, stable interspecific differences in reflectance parameters were identified across all studied weed species within a consistent spectral range. These differences manifest not only in varying reflectance values but also in contrast metrics and spectral bandwidth. Such variation provides the foundation for subsequent weed species classification based on distinct spectral signatures and corresponding plant spectral profiles.

### 2.3. Classification Model: Machine Learning Algorithms

Classification models were trained on hyperspectral images of nine weed species using five machine learning algorithms: Random Forest, Neural Network, SVM, Maximum Entropy, and SIMCA. These algorithms differ in their sensitivity to within-class spectral variability.

**Figure 14:**
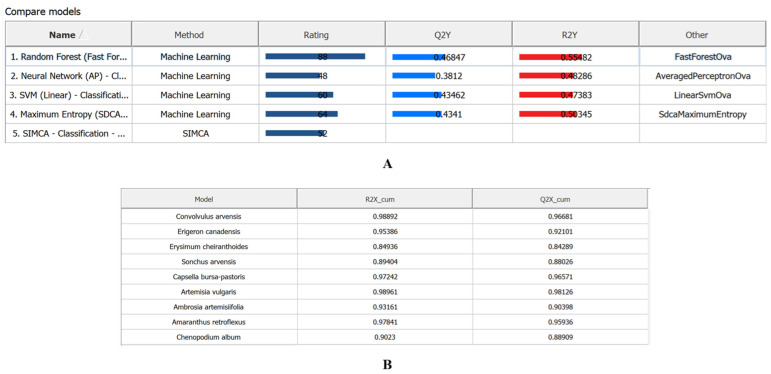
Algorithm performance comparison based on integral rating, coefficient of determination (R^2^Y), and predictive ability (Q^2^Y): (**A**)—model statistics for Random Forest, Neural Network, Maximum Entropy, and SVM; (**B**)—model statistics for SIMCA.

**Figure 15:**
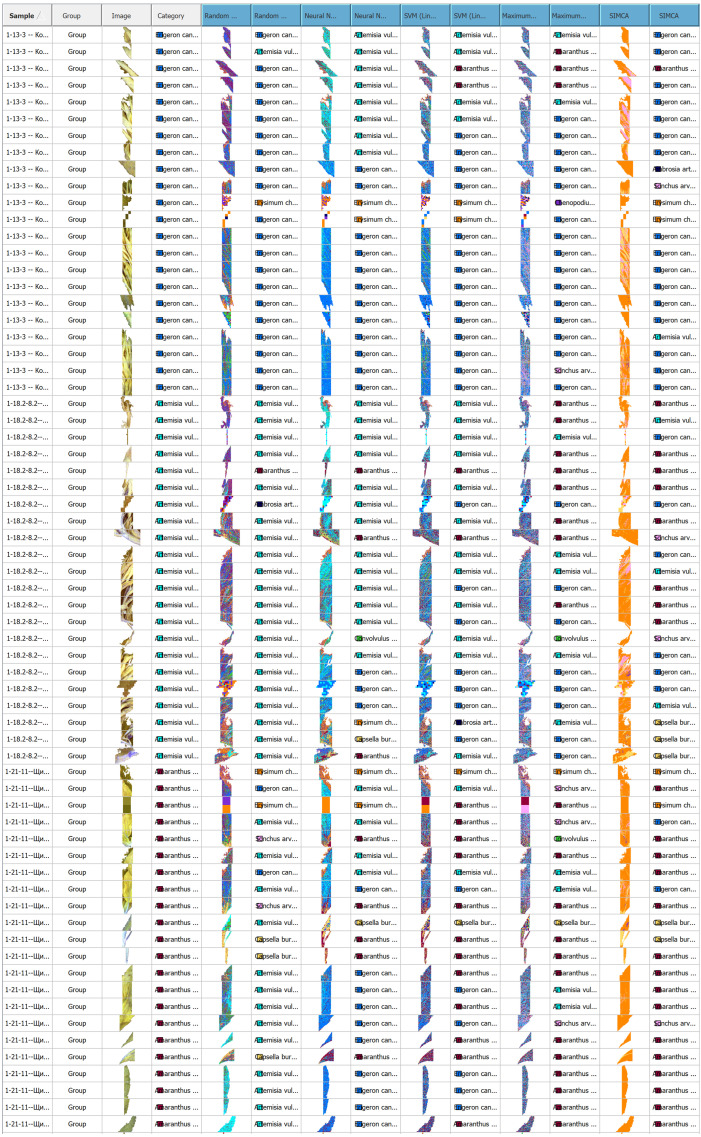
Results of automated classification of weed vegetation areas using machine learning algorithms.

Based on performance metrics, Random Forest proved most effective in terms of explained variance (R^2^Y) and predictive ability (Q^2^Y), demonstrating high classification accuracy with rapid training times. Neural Network and SVM showed lower stability and overall performance rankings. SIMCA was the least effective for weed species classification, despite relatively high R^2^Y and Q^2^Y values for individual species. Classification errors were primarily attributable to spectral signature overlap in morphologically similar plant structures (Figure 14 and Figure 15).

**Figure 16:**
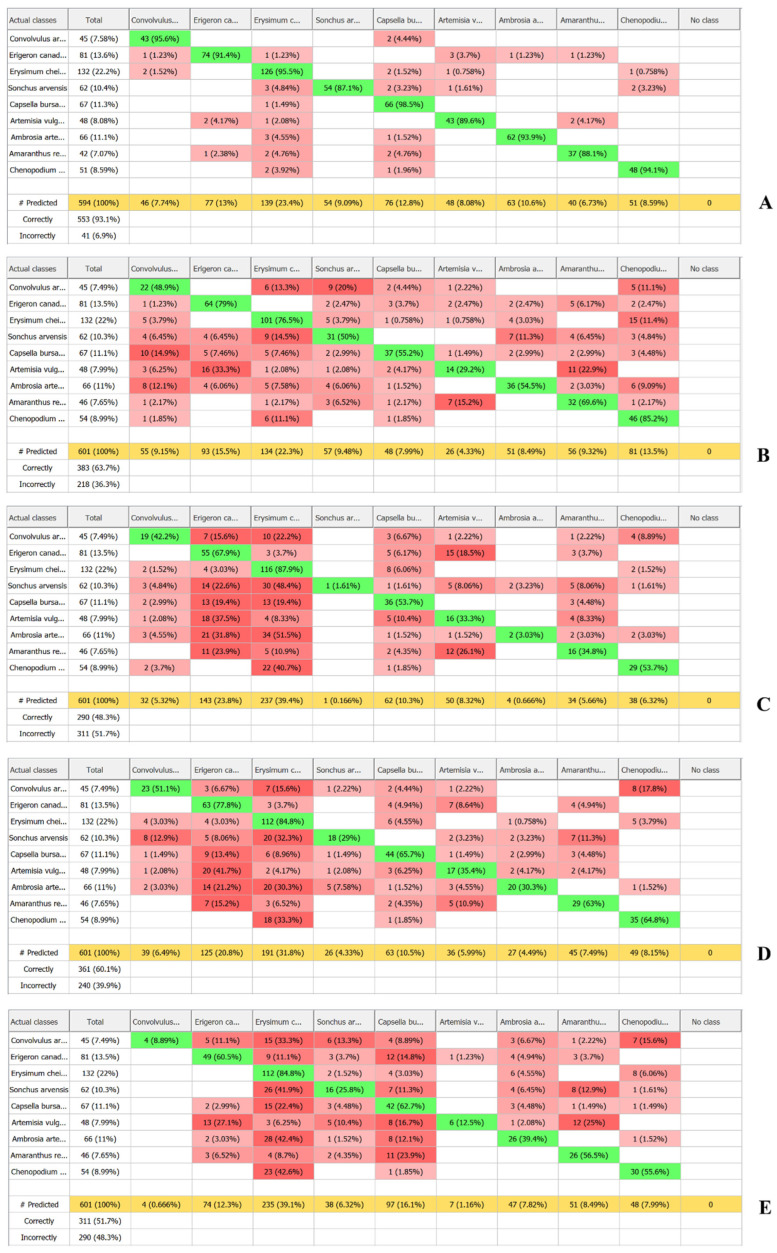
Confusion matrix ((**A**) Random Forest, (**B**) Maximum Entropy, (**C**) Neural Network, (**D**) SVM, (**E**) SIMCA): green—correctly classified samples; bright red—largest proportion of incorrectly classified samples; pale red—smallest proportion of incorrectly classified samples.

**Figure 17:**
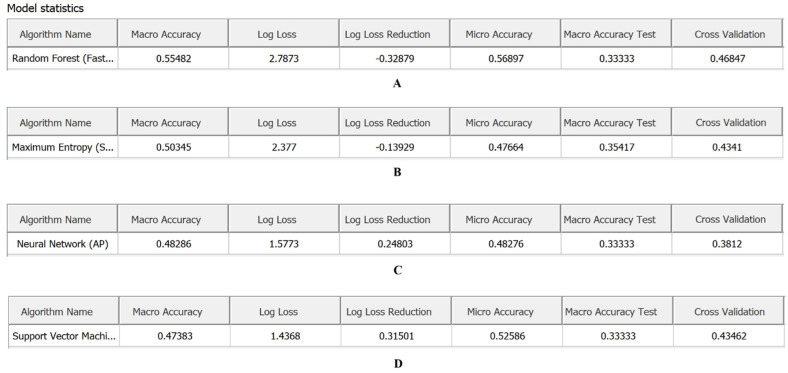
Model statistics ((**A**) Random Forest, (**B**) Maximum Entropy, (**C**) Neural Network, (**D**) SVM).

**Figure 18:**
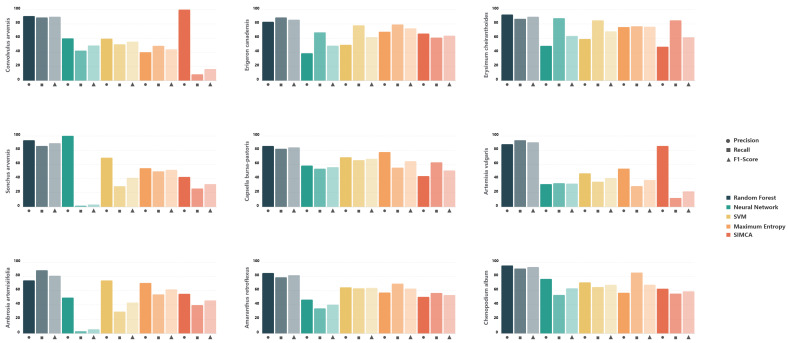
Distribution of key classification metrics (Precision, Recall, F1-score) for weed species.

The confusion matrix (Figure 16) confirmed that Random Forest achieved the most accurate and reliable classification. Random Forest correctly classified 93.5% of samples, with an error rate of 6.9%. Most categories showed high Precision and Recall values (87.1–100%), reflected in consistently high F1-scores (89.6–96.1%). The most robust classifications were achieved for *Ambrosia artemisiifolia* (F1 = 96.1%), *Convolvulus arvensis* (F1 = 94.5%), and *Chenopodium album* (F1 = 94.1%). Slightly lower classification stability was observed for *Artemisia vulgaris* (F1 = 89.6%) and *Amaranthus retroflexus* (F1 = 90.2%), indicating spectral similarity between these species and other weeds, which reduced class assignment accuracy. The Log loss value for Random Forest reflects greater confidence in classification decisions, with this algorithm demonstrating superior class interpretability and more balanced classification across categories (Figure 16A, Figure 17A and Figure 18; Table 5).

The remaining algorithms showed lower classification accuracy. Maximum Entropy achieved 63.7% overall accuracy, with F1-scores ranging from 37.8% (*Artemisia vulgaris*) to 79.9% (*Erysimum cheiranthoides*), indicating substantial performance variation across species. The highest correct identification rates were observed for *Chenopodium album* (P = 85.2%, F1 = 68.1%), *Erysimum cheiranthoides* (P = 76.5%, F1 = 75.9%), *Erigeron canadensis* (P = 79%, F1 = 73.6%) and *Amaranthus retroflexus* (P = 69.6%, F1 = 62.7%), suggesting greater spectral uniqueness for these taxa. *Artemisia vulgaris* showed minimal Recall (29.2%) and the lowest F1-score (37.8%), likely due to broad intraspecific spectral variability and overlap with other *Asteraceae* species. Maximum Entropy demonstrated moderate classification capability, with Macro accuracy of 0.50 and Micro accuracy of 0.47, indicating limited ability to distinguish categories consistently, particularly under imbalanced class distributions. The high Log loss value (2.37), combined with negative Log loss reduction (−0.13), reflects poor probabilistic predictions and minimal improvement over baseline models (Figure 16B, Figure 17B and Figure 18; Table 5).

The Neural Network algorithm showed the lowest overall classification accuracy (48.3%) and the greatest performance instability. While high Precision was achieved for *Erysimum cheiranthoides* (87.9%) and *Erigeron canadensis* (67.9%), certain species—notably *Sonchus arvensis* (1.61%) and *Ambrosia artemisiifolia* (3.03%)—exhibited very poor classification. This instability is reflected in the wide F1-score range, from 3.17% (*Sonchus arvensis*) and 5.71% (*Ambrosia artemisiifolia*) to 62.9% (*Erysimum cheiranthoides*). The algorithm proved sensitive to training set size and balance, with evidence of overfitting for certain classes. The Log loss reduction value (0.24) indicates that the model failed to produce stable probability distributions, possibly due to limited training data or suboptimal network architecture for this specific application (Figure 16C, Figure 17C and Figure 18; Table 5).

The SVM achieved 60.1% overall classification accuracy with relatively balanced performance across categories. Compared to other algorithms, SVM performed well for *Erysimum cheiranthoides* (84.8%), *Erigeron canadensis* (77.8%), and *Capsella bursa-pastoris* (65.7%), with corresponding F1-scores of 69.3%, 62.2%, and 67.7%. Macro accuracy (0.47) and Micro accuracy (0.52) suggest moderate capability in recognising both individual classes and overall observations. SVM exhibited the lowest Log loss (1.43) and the highest Log loss reduction (0.31), indicating improved model performance compared to other algorithms. However, this may also reflect insufficient calibration of probabilistic predictions. SVM required the longest computation time, and combined with its moderate accuracy, this limits its scalability for large datasets or operational deployment (Figure 16D, Figure 17D and Figure 18; Table 5).

SIMCA demonstrated limited classification capability, with overall accuracy of 51.7%. Higher performance was observed for *Erysimum cheiranthoides* (84.8%) and *Capsella bursa-pastoris* (62.7%), while *Convolvulus arvensis* (8.89%) and *Artemisia vulgaris* (12.5%) showed extremely low Precision values. These results highlight SIMCA’s limited suitability for weed species classification tasks (Figure 16E and Figure 18; Table 5).

A comparison of machine learning algorithms shows that Random Forest offers the optimal balance among accuracy, interpretability, and computational efficiency. Random Forest best matched the requirements of weed species classification, due both to the characteristics of the input data and the structural properties of the algorithm itself. The high classification accuracy achieved by Random Forest stems from its effective identification of nonlinear relationships between spectral features and plant taxonomy. A key advantage of Random Forest in this context is its robustness to limited training sample sizes and imbalanced class distributions.

## 3. Discussion

### 3.1. Interpretation of Statistical Indicators

Analysis of species-specific statistical parameters revealed that each weed species possesses distinctive spectral identifiers. Variability in reflectance coefficients, contrast values, and spectral bandwidth confirms the existence of stable optical differences between species.

The mean reflectance parameter (μ) indicates that higher reflectance in certain species (*Capsella bursa-pastoris*, *Chenopodium album*) is associated with leaf-level reflectance properties, presumably driven by higher chlorophyll density and photosynthetic activity. Lower mean reflectance values in *Erysimum cheiranthoides* and *Ambrosia artemisiifolia* primarily reflect differences in pigment composition and morphology of vegetative foliar units, resulting in reduced above-ground biomass reflectance.

Standard deviation and coefficient of variation analyses revealed moderate intraspecific spectral variability, indicating that individual morphological structures within each species maintain stable spectral responses. A notable trend emerged regarding ΔR amplitude: the highest values were observed predominantly in species with larger leaf blade areas. This suggests that foliar tissues contribute substantially to the integrated spectral signal of each plant profile.

The spectral wavelength range showed that most species exhibit full-range responses from 500 to 780 nm, encompassing both VIS and NIR regions. Narrowed spectral bandwidth (550–780 nm) in roots and basal structures corresponds to the limited reflectance properties of these tissues and their reduced contribution to overall plant spectral signatures.

Interspecific differentiation stability is evident from normalised contrast and spectral bandwidth values: increases in the former consistently accompany increases in the latter. *Erysimum cheiranthoides*, *Sonchus arvensis*, *Capsella bursa-pastoris*, and *Ambrosia artemisiifolia* exemplify this pattern. This combination of spectral parameters may serve as optimal spectral indicators for remote—including proximal—species recognition based on spectral characteristics.

Thus, integrating spectral distribution patterns with reflectance metrics enables the use of both intraspecific and interspecific variations for weed classification using algorithms designed for crop monitoring models. These findings also confirm the importance of accounting not only for spectral properties but also for specific morphological structures when developing spectral profiles for recognition and differentiation models.

### 3.2. Spectral Characteristics of Weeds

HSI captures individual plant pixels and converts them into spectral information that reflects the plant’s physiological state and biochemical composition. In the NIR and SWIR ranges, reflectance depends on internal structure and water content, enabling assessment of plant physiological condition and species identification against background vegetation.

The VIS region (400–700 nm) corresponds to light absorption by photosynthetic pigments, while in the NIR range (700–1100 nm), plants reflect incident radiation. Reflectance intensity depends on plant structure (leaf blade area, tissue thickness, spongy mesophyll development, water balance, nitrogen content) and particularly on pigment composition and function—primarily chlorophylls and carotenoids. Stressed or wilting plants exhibit lower reflectance intensity compared to healthy, vigorous specimens. Most samples showed significantly higher reflectance in the NIR range (780 nm) than in the VIS region, though spectral curves varied across different plant zones and tissues (Table 6).

Pigment absorption and leaf structure create characteristic spectral features. Different pigment complexes (antennas of photosynthetic systems I and II) exhibit distinct spectral properties and peak absorption values associated with chlorophyll and its interactions within thylakoid membranes, as documented extensively in the literature [46,47]. Pigments absorb light when photon energy matches the energy levels of π-electrons in the molecule, triggering electronic transitions. Our data confirm that chloroplasts absorb light in the visible range (400–700 nm). Low reflectance in the blue (500 nm) and red (690 nm) regions results from strong chlorophyll absorption. Increased reflectance occurs in the green region (500–550 nm) and the 700–780 nm zone. The red edge region (690–740 nm) marks the transition from the visible to near-infrared spectrum. Red edge position and shape are sensitive to chlorophyll content. The gradual intensity decrease and spectral trough near 690 nm reflect chlorophyll light absorption, while the sharp reflectance increase around 700 nm results from NIR radiation scattering by plant structures—consistent with findings from other studies [38,46,47,48]. Chloroplasts exhibit characteristic fluorescence peaks (around 550 nm) reflecting chlorophyll energy transitions in photosystems I and II. Leaf veins showed lower reflectance due to reduced water content from slight sample desiccation.

Other leaf components exert less influence on spectra within this range. Roots and basal zones reflect intensively in both VIS and NIR regions due to the absence of chlorophyll and mesophyll structure, and predominance of weakly absorbing compounds (lignin, suberin). Amyloplasts reflect radiation uniformly across 400–780 nm. Plant parts containing amyloplasts generally show higher reflectance than green tissues. *Capsella bursa-pastoris* flowers are white and pigment-free, explaining their higher VIS reflectance compared to NIR.

Carotenoids function as photoprotective pigments in chloroplasts and as pigments in specific plant parts, absorbing light up to 500–550 nm. Flowers contain xanthophylls and carotenes (primarily lutein, β-carotene, violaxanthin, neoxanthin, zeaxanthin, antheraxanthin). Carotenoids possess long conjugated double-bond chains (C = C) and absorb high-energy photons; their π-electron system absorbs short-wavelength, high-energy light, resulting in low blue-region reflectance. Weaker absorption compared to chlorophyll produces relatively higher reflectance in the visible spectrum, such as in the yellow region observed in *Erysimum cheiranthoides*. No absorption is detectable in other spectral regions, with reflectance increasing in the NIR range. Flower colouration serves adaptive functions: pigments protect photosystem II from photodamage, suppress chlorophyll triplet states, act as antioxidants, and attract pollinators.

**Figure 19:**
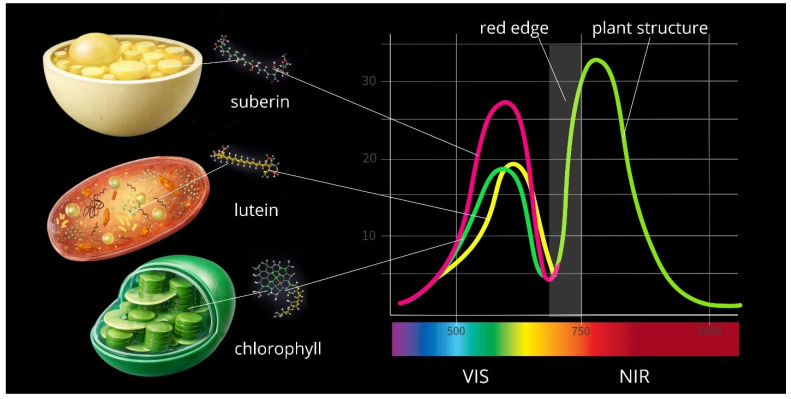
Relationship between spectral regions (VIS, NIR) and pigment composition of plant tissues.

Figure 19 summarises the relationships between spectral characteristics and pigment composition. Infrared radiation is reflected by plant structures, while curve shape and reflectance intensity in the visible spectrum are influenced by pigments.

Overall, light-coloured root zones exhibit the highest reflectance. Bright flowers reflect less radiation than roots but more than green plant tissues. NIR reflectance is determined primarily by leaf structural properties.

**Figure 20:**
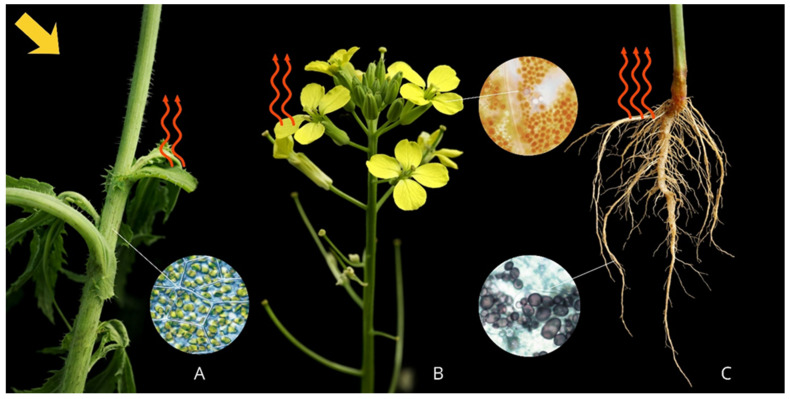
Variation in spectral reflectance across different weed plant parts ((**A**) leaves, stem; (**B**) flowers; (**C**) root, root collar; two arrows—medium reflectance, three arrows—high reflectance).

Generalised reflectance intensity patterns for different weed plant parts are presented in Figure 20.

Each spectral curve encodes information about unique structural features of weed species. Similar curve shapes were observed for *Capsella bursa-pastoris*, *Amaranthus retroflexus*, and partially *Convolvulus arvensis*, with high VIS reflectance attributable to smooth epidermal tissue containing few light-absorbing pigments. Other weeds showed higher NIR reflectance. In the overall spectral comparison, *Convolvulus arvensis* and *Erysimum cheiranthoides* exhibited the lowest reflectance coefficients, associated with reduced intercellular spaces and dense palisade parenchyma. Higher reflectance in *Sonchus arvensis* and *Chenopodium album* resulted from light scattering by trichomes.

Due to morphological differences, weed spectra possess characteristics that distinguish them from crops and other vegetation types in terms of curve shape and reflectance values at key wavelengths. The most significant wavelength regions are 550–600 nm, 690–700 nm, and 750 nm. In the VIS region (500–700 nm), weeds show variable absorption intensity, manifested as modified spectral curve shapes where reflectance minima may be less pronounced or shifted, and contrast between green and red regions may be reduced or enhanced compared to crops. In the red edge region (700 nm), weed species exhibit significant differences in transition position and steepness from absorption to high reflectance. This region is characterised by more gradual, less stable reflectance increases and interspecific shifts in the wavelength of maximum gradient. In the NIR region (700–780 nm), weeds differ in reflectance plateau level and shape; unlike crops grown in relatively homogeneous agrocenoses, weed spectra demonstrate high interspecific dispersion of reflectance values. However, all weed species maintain common curve configuration characteristics, with low peaks in the VIS region and maximum values in the NIR zone.

The spectral specificity of weeds arises from both overall curve shapes for each species and internal differences between plant parts within individual specimens. These patterns underpin the application of machine learning methods capable of capturing nonlinear spectral differences during weed identification. Random Forest operates through sequential logical rules, requires limited training examples, and identifies localised threshold differences in spectra. Each algorithm node selects the feature (wavelength) and threshold that maximally separates classes. Species differences often manifest in narrow spectral intervals, and Random Forest can utilise specific wavelengths as discriminating features. Each tree trains on random object subsamples, using random feature subsets at each node, with final decisions based on ensemble voting. However, method effectiveness may decrease in cases of high spectral similarity between classes and limited training sample size. Maximum Entropy suits limited data scenarios but is not optimised for multiclass spectral classification. Neural Networks model nonlinear relationships between input spectra and classes but require extensive data, leading to underfitting with small samples. SVM is sensitive to noise and class imbalance. SIMCA constructs individual PCA models for each class but performs poorly with significant category overlap. Consequently, Random Forest enables identification of key spectral ranges and is better suited for automated weed identification, particularly under sample size constraints.

Despite the high overall accuracy of the Random Forest model, analysis of the confusion matrix revealed persistent misclassifications between certain species pairs. Such systematic confusion indicates partial overlap of their spectral characteristics within specific wavelength ranges, which reduces class separability for the classification algorithm.

*Sonchus arvensis* was most frequently misclassified as Erysimum cheiranthoides (4.84%), *Capsella bursa-pastoris* (3.23%), and *Chenopodium album* (3.23%). All of these species exhibit light-coloured areas with increased reflectance, leading to partial overlap of spectral features despite differences in the overall shape of their spectral curves. In *Sonchus arvensis*, higher reflectance in the NIR region is associated with the presence of trichomes. In *Capsella bursa-pastoris* and *Erysimum cheiranthoides*, increased reflectance in the VIS region results from light-coloured flowers, while in *Chenopodium album* it is caused by the light basal zone. In hyperspectral data, such bright elements are encoded with similar values, thereby reducing the model’s discriminative capacity. White or light-coloured areas generate a similar mechanism of diffuse scattering. From the sensor’s perspective, this appears as an increased reflectance coefficient across a broad wavelength range. The model may interpret different morphological structures as the same spectral feature, grouping plants by overall reflectance level, which leads to class overlap and minor classification errors.

*Artemisia vulgaris* was most frequently classified as *Erigeron canadensis* (4.17%) and *Amaranthus retroflexus* (4.17%). As in the previous case, despite differences in spectral shape, these species share similar leaf morphology and pigment composition. The overlap of these characteristics leads to class confusion.

*Amaranthus retroflexus* was misclassified as *Erysimum cheiranthoides* (4.76%) and *Capsella bursa-pastoris* (4.76%). These species are characterised by similar spectral curve shapes with a pronounced red edge around 700 nm, as well as reflectance levels in the VIS region that are equal to or higher than those in the NIR region. In this case, spectral differences are insufficiently pronounced for confident separation due to similarities in morphology and pigment reflectance properties, which explains the recurring classification errors under similar spectral characteristics.

Overall, the Random Forest algorithm demonstrates high efficiency when working with high-dimensional spectral data and is capable of identifying nonlinear threshold differences at specific wavelengths, making it robust even with a relatively limited training dataset. Nevertheless, classification accuracy largely depends on the degree of separability of species spectral signatures in key wavelength ranges. Alternative methods, such as SVM and Neural Networks, may achieve comparable accuracy with larger datasets and enhanced preprocessing, but they require additional optimisation and validation.

### 3.3. Study Limitations and Generalisability

The current study included plant samples from multiple phenological stages within a single geographic region and growing season, allowing reliable identification of spectral features across developmental stages. It remains to be determined how well the models perform on data collected under different locations, seasons, or field conditions. Environmental factors—such as changes in illumination, soil conditions, plant water status, or senescence—can modify spectral signatures and cause domain shifts, potentially lowering classification accuracy. While the plant-level train/test split ensures that the models generalise to unseen plants within the same sample set, further validation on independent datasets collected under varied field conditions and across multiple growing seasons would be valuable. Additionally, exploring data augmentation or domain adaptation strategies in future work could help improve model robustness and facilitate their practical application in precision agriculture.

## 4. Materials and Methods

### 4.1. Study Objects and Sampling Sites

**Figure 21:**
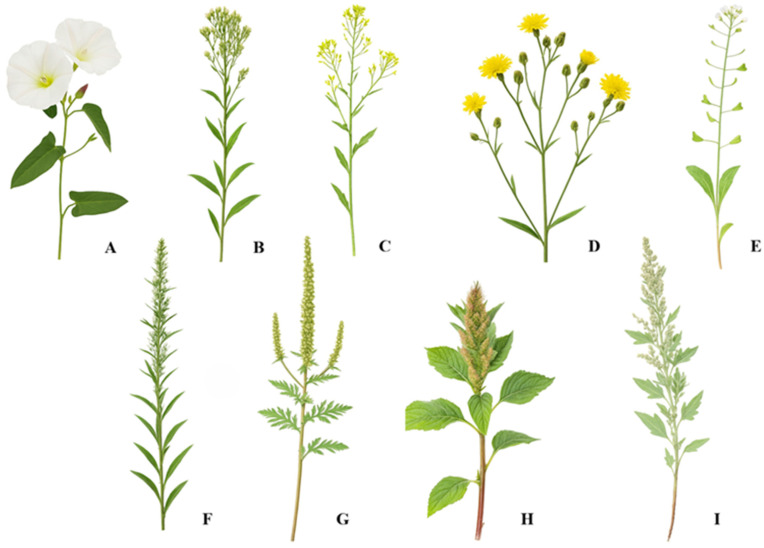
Study objects ((**A**) *Convolvulus arvensis*, (**B**) *Erigeron canadensis*, (**C**) *Erysimum cheiranthoides*, (**D**) *Sonchus arvensis*, (**E**) *Capsella bursa-pastoris*, (**F**) *Artemisia vulgaris*, (**G**) *Ambrosia artemisiifolia*, (**H**) *Amaranthus retroflexus*, (**I**) *Chenopodium album*).

This study focused on nine weed species commonly found in spring wheat (*Triticum aestivum* L.) agrocenoses: *Convolvulus arvensis* (L., 1753), *Erigeron canadensis* (L., 1753), *Erysimum cheiranthoides* (L., 1753), *Sonchus arvensis* (L., 1753), *Capsella bursa-pastoris* (Medik., 1792) (syn. *Thlaspi bursa-pastoris* (L., 1753), *Artemisia vulgaris* (L., 1753), *Ambrosia artemisiifolia* (L., 1753), *Amaranthus retroflexus* (L., 1753), *Chenopodium album* (L., 1753) (Figure 21).

Plant samples were collected in 2025 from spring wheat fields in the main grain-producing areas of northeastern Kazakhstan (Pavlodar Region; 52.2–52.5° N, 76.8–77.1° E). Sampling was timed to key phenological stages of the wheat crop according to the BBCH scale (Biologische Bundesanstalt, Bundessortenamt und Chemische Industrie): 25–35, 39–59 и 61–75. This standardised scale enabled consistent staging of wheat development across sampling events.

The sampling sites were located in typical steppe agrolandscapes with flat terrain and a strongly continental climate—characterised by hot, dry summers and cold winters. The growing season was moderately warm (mean daily temperatures ranged from +13 to +22 °C) with uneven precipitation: early-season deficits (15–25 mm) followed by above-average rainfall in the second half of summer (65–70 mm). Soils were predominantly dark chestnut and medium-loamy chestnut types, with organic matter content of 2.5–3.0%, pH between 7.3 and 7.6, and calcareous soils.

A total of 225 individual plants were collected across nine weed species, with 25 plants per species. From each plant, multiple regions of interest were manually selected from different morphological parts (e.g., leaves, stems, flowers, roots) to capture the full range of intra-species spectral variability.

A hierarchical two-tier validation strategy was employed: the 225 plants were randomly split into training (70% per species) and testing (30% per species) sets, ensuring testing plants were completely independent. ROIs from the same plant were never included in both sets.

### 4.2. Hyperspectral Imaging

Laboratory studies were conducted at the Biological Research Laboratory of Toraighyrov University (Pavlodar, Kazakhstan).

#### 4.2.1. Sample Preparation for Hyperspectral Imaging

Before image acquisition, custom calibration panels with flat, matte surfaces were prepared to minimise specular reflection. Each plant sample was positioned individually to prevent overlapping of vegetative parts and to facilitate accurate segmentation. The camera was positioned at a distance of 30–40 cm from the samples. The weed samples were fixed in their natural state to preserve morphological integrity and enable preliminary calibration prior to imaging using dark and white reference standards (Spectralon^®^ with 99% diffuse reflectance across the VNIR spectrum due to its fluoropolymer material; Labsphere, Inc., North Sutton, NH, USA) [49]. This step reduced noise and improved the accuracy of reflectance measurements.

ROI selection was guided by PCA-based variance scatter plots and max variance images in Pixel Explore, which helped identify spectrally homogeneous and informative tissue regions while avoiding shadows, specular reflections, and damaged areas. A total of 601 ROIs were extracted: *Erysimum cheiranthoides* (132 ROIs), *Erigeron canadensis* (81), *Capsella bursa-pastoris* (67), *Ambrosia artemisiifolia* (66), *Sonchus arvensis* (62), *Chenopodium album* (54), *Artemisia vulgaris* (48), *Convolvulus arvensis* (45), *Amaranthus retro-flexus* (46). On average, 2–3 ROIs were collected per plant, each containing at least 50 pure pixels.

#### 4.2.2. Hyperspectral Image Acquisition and Processing

##### Technical Parameters and Imaging Mode

Hyperspectral imaging was conducted using a FigSpec^®^ FS-13 VNIR camera (CHNSpec Technology, Hangzhou, China) operating in push-broom scanning mode across the 400–1000 nm spectral range. Key specifications include: transmission diffraction grating, 1200 spectral channels, spectral resolution of 2.5 nm, spatial resolution of 1920 pixels per line, and a pixel size of 5.86 µm, CMOS detector (providing low noise and high sensitivity). Images were captured at 128 frames per second in full spectral load mode. These parameters enabled comprehensive spectral analysis across the full range, capturing the morphological diversity of the weed species under study [50].

##### Image Processing and Preparation for ML-Based Classification

Raw hyperspectral data cubes were processed using Breeze software (v2024.2.0, Umeå, Sweden). Spectral signatures were extracted from different morphological structures of each specimen. Each image formed a three-dimensional hypercube combining two-dimensional spatial data with high-resolution spectral information. Both spectral and radiometric calibrations were applied. Correction coefficients were derived from dark and white reference images. Relevant pixels were isolated and specular artefacts removed using spatial masking with ROI tools and binarisation methods based on spectral homogeneity. Clustering and morphological filtering algorithms were employed where appropriate. Raw spectral data were smoothed using the built-in moving average function in Breeze software. Following smoothing, Standard Normal Variate (SNV) transformation was applied to correct for multiplicative scatter effects and baseline shifts. No band trimming was performed; the full 400–1000 nm range was retained.

#### 4.2.3. Interactive Spectral Analysis Using PCA and Pixel Explore

Pixel Explore was used to interactively link regions of interest in hyperspectral images to their corresponding positions on PCA plots. Raw spectrum plots, variance scatter plots, and variance-scaled hyperspectral images were generated to visualise data structure. Raw spectrum extraction was performed for selected areas across the full spectral range. Variance scatter plots revealed pixel distributions across principal components (t [1]–t [6]), with the first two components (t [1] and t [2]) retained as the most informative. PC1 captured the largest spectral differences and was used as a primary axis for grouping spectrally similar signatures. PC2 reflected morphological and surface texture variability. Point density was colour-coded with red indicating areas of highest pixel density. Max variance images were used to project hypercube data onto channels exhibiting maximum spectral variation, facilitating identification of the most informative zones within each sample.

#### 4.2.4. ML-Based Species Identification and Classification

To enable automated weed identification and differentiation, classification models were trained using several machine learning algorithms. Feature vectors were constructed by combining spectral signatures with optimised feature selection. PCA and Sample model were applied to reduce dimensionality, suppress background pixels, capture major sources of variance, and also for ROI extraction. The Sample model refers to a supervised sample-based segmentation approach in which representative spectral samples of each class were selected to generate reference spectral profiles. These profiles were subsequently used to identify spectrally similar regions prior to classification.

**Figure 22:**
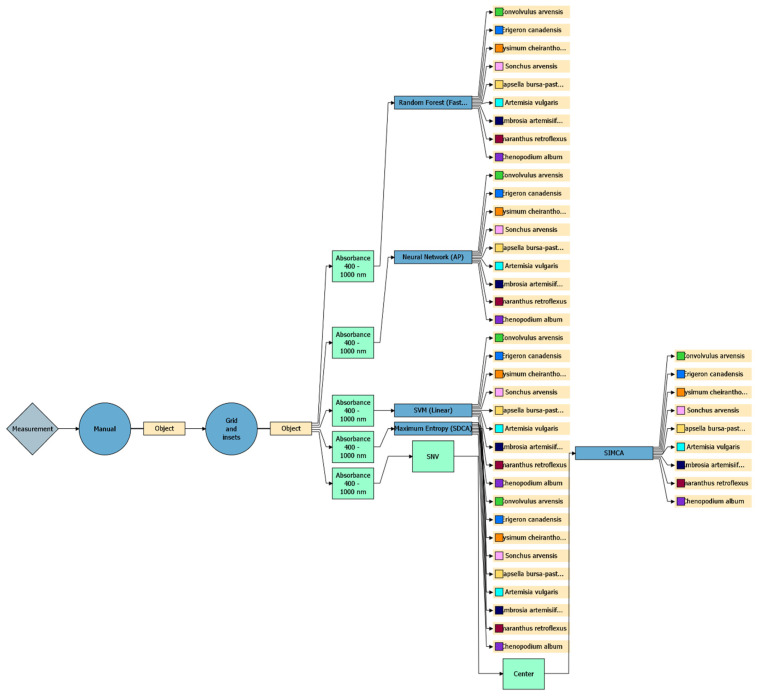
Workflow of machine learning algorithms applied for intelligent weed species identification and differentiation.

Five machine learning algorithms were evaluated (Figure 22):

(1) Random Forest (Fast Forest)—an ensemble of decision trees; robust to noise and capable of modelling nonlinear relationships. Capable of integrating both visual and spectral features during training [51];
(2) Maximum Entropy (SDCA)—a linear logistic regression model based on the principle of Maximum Entropy; suitable for large-scale multiclass problems [52];
(3) Neural Network (AP)—performs linear class separation; may oversmooth complex nonlinear dependencies [53];
(4) SVM—a linear classifier scalable to high-dimensional feature spaces and supports retraining. Performance may be constrained by complex, nonlinear data structures [54];
(5) SIMCA—classifies samples based on their proximity to PCA models constructed for each class; leverages within-class variability and performs well with limited training data [55].

All machine learning models were implemented using software with default optimised algorithms. Key hyperparameters:

(1) Random Forest: 100 trees, unrestricted depth, 11 features per split;
(2) SVM: Linear kernel, C = 1.0;
(3) Neural Network: Single hidden layer, neurons auto-selected, logistic activation;
(4) Maximum Entropy (SDCA): λ = 0.001;
(5) SIMCA: PCA per class with the number of components chosen to capture the dominant class structure (PC1-PC2).

ROI segmentation was performed manually using the mesh function to capture heterogeneous tissue regions, resulting in 601 sample regions. Hyperparameter tuning was integrated into 5-fold cross-validation via grid search on the 420 training ROIs to assess internal stability, while 181 ROIs from testing plants were reserved exclusively for final evaluation (plant-wise split). All hyperspectral images were obtained under standardised laboratory conditions with consistent lighting and fixed imaging protocols to minimise measurement variability, despite plants being collected from multiple fields and phenological stages. All spectral data were processed within the 400–1000 nm VNIR range, and model performance was evaluated using Precision, Recall, and F1-score metrics.

### 4.3. Statistical Data Processing

Classification reliability and accuracy were assessed using descriptive statistics and variance analysis of spectral features. The formulas and corresponding interpretations for all calculated metrics are presented in Table 7.

The derived statistical parameters were used to build classification models for weed identification and monitoring. Scaling and field deployment of these predictive models are expected to improve the effectiveness of crop protection strategies against weed infestation.

## 5. Conclusions

This study explored the application of hyperspectral imaging for weed species identification. Spectral characteristics of nine weed species (*Convolvulus arvensis*, *Erigeron canadensis*, *Erysimum cheiranthoides*, *Sonchus arvensis*, *Capsella bursa-pastoris*, *Artemisia vulgaris*, *Ambrosia artemisiifolia*, *Amaranthus retroflexus*, *Chenopodium album*) revealed both similarities—attributable to comparable ecological growing conditions—and differences arising from morphological variation and pigment composition.

The spectral specificity of weeds arises from the combined characteristics of whole-plant spectral curves for each species and internal differences between plant parts within individual specimens. Differences between species’ spectra reflect morphological diversity. Reflectance in the VIS region depends on pigment composition and absorption capacity. High reflectance originates from zones dominated by amyloplasts (basal regions, white flowers), with progressively lower reflectance from carotenoid-containing tissues (yellow flowers) and chlorophyll-containing tissues (stems, leaves), where light absorption supports photosynthetic activity. In the NIR region, reflectance coefficients reflect the integrated structural features of each species. Both reflectance intensity and spectral curve shapes reveal adaptive characteristics of weed species.

Spectral data were analysed using five machine learning algorithms: Random Forest, Maximum Entropy, Neural Network, SVM, and SIMCA. Model performance was evaluated using class-specific and overall accuracy metrics. For weed species classification based on limited hyperspectral datasets, Random Forest proved most suitable, achieving 93.5% classification accuracy. Its high effectiveness stems from the hierarchical threshold rule system that sequentially separates classes based on the most informative features. This approach reduces overfitting risk and delivers more stable results than probabilistic methods when spectral overlap between species occurs.

The combination of hyperspectral imaging and machine learning demonstrates strong potential for accurate weed identification, particularly when employing the Random Forest algorithm, whose robustness to imbalanced and limited datasets constitutes a key advantage. This approach opens possibilities for integrating intelligent agroecological monitoring and weed management systems into precision agriculture practice, enabling reduced herbicide application and consequent lowering of environmental impact on crop stands. Looking forward, expanding weed spectral profile databases, adapting ML models to diverse ecological zones and type of crops and integrating with UAV platforms could further enhance the accuracy and speed of agricultural monitoring, thereby promoting sustainable agricultural development and management.

## Figures and Tables

**Table 1:** Explained variance per principal component (PC1–PC6) from spectral data analysis.

Weed Species	PC1	PC2	PC3	PC4	PC5	PC6
	%					
*Convolvulus arvensis*	79.50	9.82	1.48	0.458	0.186	0.088
*Erigeron canadensis*	79.90	11.90	1.16	0.581	0.179	0.049
*Erysimum cheiranthoides*	78.50	7.40	0.662	0.403	0.23	0.076
*Sonchus arvensis*	85.60	4.40	0.419	0.402	0.142	0.053
*Capsella bursa-pastoris*	91.20	5.43	0.738	0.185	0.072	0.049
*Artemisia vulgaris*	76.30	14.80	1.68	0.451	0.171	0.044
*Ambrosia artemisiifolia*	83.70	6.90	0.674	0.347	0.125	0.045
*Amaranthus retroflexus*	79.10	13.00	1.70	0.486	0.179	0.052
*Chenopodium album*	81.60	9.13	1.70	0.266	0.117	0.061

**Table 2:** PCA variance statistics.

Measured Parameter	PC1	PC2	PC3	PC4	PC5	PC6
	%					
Explained Variance (%)	81.71	9.2	1.14	0.40	0.16	0.06
Cumulative Variance (%)	81.71	90.91	92.05	92.45	92.61	92.67
Eigenvalue (if total variance = 1)	0.817	0.092	0.011	0.004	0.002	0.001

**Table 3:** Spectral characteristics of weeds: reflectance parameters.

№	Species	Part	Reflection Coefficient (%)		Mean Reflectance	Standard Deviation	Coefficient of Variation	Median	Rate of Change	Delta Reflectance
			min	max	μ	σ	CV	Me	R	ΔR
1	*Convolvulus arvensis*	General	5	15	22.6	2.1	10.1	22.6	11.3	16.8
		Body	5	45						
		Root	20	35						
		Root collar	25	30						
		Leaf	10	31						
		Stem	20	30						
2	*Erigeron canadensis*	General	8	23	20.9	2.8	13.5	20.9	14.8	22.3
		Body	5	45						
		Root	19	30						
		Leaf	7	30						
3	*Erysimum cheiranthoides*	General	6	16	20.5	2.2	11.1	20.5	11.2	17.4
		Body	5	40						
		Flower	20	35						
		Stem	18	30						
		Leaf	10	25						
4	*Sonchus arvensis*	General	11	25	21.1	2.2	10.6	21.1	10.9	17.0
		Body	3	35						
		Root	19	30						
		Stem	15	30						
		Leaf	15	28						
5	*Capsella bursa-pastoris*	General	14	21	24.5	2.3	8.7	24.5	11.8	18.4
		Body	10	50						
		Leaf	15	45						
		Flower	22.5	30						
		Stem	15	22.5						
6	*Artemisia vulgaris*	General	8	19	22.5	2.6	11.1	22.5	13.8	20.7
		Body	10	55						
		Root collar	15	40						
		Root	15	30						
		Stem	15	25						
		Leaf	10	28						
7	*Ambrosia artemisiifolia*	General	6	21	18.0	2.4	13.6	18.0	12.2	19.0
		Body	3	35						
		Leaf	15	29						
		Stem	10	25						
8	*Amaranthus retroflexus*	General	9	26	22.6	2.3	10.5	22.6	12.5	18.5
		Body	7.5	43						
		Root	20	40						
		Stem	20	25						
		Leaf	10	25						
9	*Chenopodium album*	General	14	25	24.8	2.5	9.3	24.8	12.5	19.4
		Body	7	55						
		Leaf	22.5	45						
		Stem	22	25						
		Root	10	22.5						

**Table 4:** Spectral characteristics of weeds: wavelength parameters.

№	Species	Part	Wavelength (nm)		Spectral Bandwidth	Contrast Ratio	Relative Bandwidth	Normalised Contrast
			min	max	SB	C	B_rel_	C_norm_
1	*Convolvulus arvensis*	General, Body	500	780	246.7	1.465	0.188	0.316
		Root, Root collar, Leaf, Stem	550	780				
2	*Erigeron canadensis*	General, Body	500	780	255.0	1.489	0.196	0.327
		Root, Leaf	550	780				
3	*Erysimum cheiranthoides*	General, Body, Flower, Stem, Leaf	500	780	280.0	1.560	0.219	0.359
4	*Sonchus arvensis*	General, Body, Root, Stem, Leaf	500	780	280.0	1.560	0.219	0.359
5	*Capsella bursa-pastoris*	General, Body, Flower, Stem, Leaf	500	780	280.0	1.560	0.219	0.359
6	*Artemisia vulgaris*	General, Body, Leaf	500	780	255.0	1.489	0.196	0.327
		Root, Root collar, Stem	550	780				
7	*Ambrosia artemisiifolia*	General, Body, Stem, Leaf	500	780	280.0	1.560	0.219	0.359
8	*Amaranthus retroflexus*	General, Body	500	780	238.0	1.453	0.184	0.309
		Root, Stem, Leaf	550	780				
9	*Chenopodium album*	General, Body, Root, Leaf	500	780	270.0	1.532	0.210	0.346
		Stem	550	780				

**Table 5:** Comparative evaluation of weed species classification quality by class based on key metrics (Precision, Recall, F1-score).

Metrics	*Convolvulus arvensis*	*Erigeron canadensis*	*Erysimum cheiranthoides*	*Sonchus arvensis*	*Capsella bursa-pastoris*	*Artemisia vulgaris*	*Ambrosia artemisiifolia*	*Amaranthus retroflexus*	*Chenopodium album*
Random Forest									
Precision	93.5	96.1	90.6	100	86.8	89.6	98.4	92.5	94.1
Recall	95.6	91.4	95.5	87.1	98.5	89.6	93.9	88.1	94.1
F1-score	94.5	93.7	93	93.1	92.3	89.6	96.1	90.2	94.1
Maximum Entropy									
Precision	40	68.8	75.4	54.4	77.1	53.8	70.6	57.1	56.8
Recall	48.9	79	76.5	50	55.2	29.2	54.5	69.6	85.2
F1-score	44	73.6	75.9	52.1	64.3	37.8	61.5	62.7	68.1
Neural Network									
Precision	59.4	38.5	48.9	100	58.1	32	50	47.1	76.3
Recall	42.2	67.9	87.9	1.61	53.7	33.3	3.03	34.8	53.7
F1-score	49.4	49.1	62.9	3.17	55.8	32.7	5.71	40	63
SVM									
Precision	59	50.4	58.6	69.2	69.8	47.2	74.1	64.4	71.4
Recall	51.1	77.8	84.8	29	65.7	35.4	30.3	63	64.8
F1-score	54.8	61.2	69.3	40.9	67.7	40.5	43	63.7	68
SIMCA									
Precision	100	66.2	47.7	42.1	43.3	85.7	55.3	51	62.5
Recall	8.89	60.5	84.8	25.8	62.7	12.5	39.4	56.5	55.6
F1-score	16.3	63.2	61	32	51.2	21.8	46	53.6	58.8

**Table 6:** Reflectance characteristics of weed species within the VNIR spectral range.

Spectral Range	Wavelength (nm)	Description
VIS	400–700	Reflectance depends on pigment composition and absorption capacity. High reflectance originates from zones dominated by amyloplasts; lower reflectance from chlorophyll-containing zones, where light is absorbed for photosynthetic activity.
NIR	700–1100	Reflectance depends on the overall plant structure or specific tissue architecture. The highest reflectance in this region originates from root and basal systems.

**Table 7:** Descriptive and variation metrics for spectral data: formulas and interpretation [56,57,58,59,60,61].

№	Metric	Formula	Interpretation
1	Explained Variance	$\mathrm{Explained}\;{\mathrm{Variance}}_{i}\left(\%\right)=$ $=\frac{\lambda_{i}}{\sum_{j=1}^{m}\lambda_{j}}\times 100\%$	Proportion of total variance explained by the *i*-th component
2	Cumulative Variance	$\mathrm{Cumulative}\;{\mathrm{Variance}}_{k}\left(\%\right)=$ $=\sum_{i=1}^{k}\frac{\lambda_{i}}{\sum_{j=1}^{m}\lambda_{j}}\times 100\%$	Cumulative proportion of variance up to the *k*-th component
3	Maximum reflectance	$R_{max}=max(R_{1},\;R_{2},\;\ldots ,R_{n})$	Upper and lower bounds of reflectance values
4	Minimum reflectance	$R_{min}=min(R_{1},\;R_{2},\;\ldots ,R_{n})$	
5	Mean reflectance	$\mu =\frac{1}{n}\sum_{i=1}^{n}R_{i}$,	Average intensity of reflected light
6	Standard deviation	$\sigma =\sqrt{\frac{1}{n}\sum_{i=1}^{n}{{(R}_{i}-\mu )}^{2}}$	Dispersion of reflectance values around the mean
7	Coefficient of variation	$CV=\frac{\sigma }{\mu }\times 100$	Degree of spectral variability
8	Rate of change	$R=\frac{R_{2}-R_{1}}{\lambda_{2}-\lambda_{1}}$	Spectral shift across adjacent wavelengths
9	Delta reflectance	$\Delta R=R_{max}-R_{min}$	Total reflectance range within the measured spectrum
10	Spectral bandwidth	$SB=\lambda_{max}-\lambda_{min}$	Width of the spectral range
11	Contrast ratio	$C=\frac{x_{max}}{x_{min}}\;\;\;\;\;\;\;\;(x_{min}\neq 0)$	Ratio between maximum and minimum spectral response
12	Relative bandwidth	$B_{rel}=\frac{x_{max}-x_{min}}{x_{max}+x_{min}}$	Normalised measure of spectral heterogeneity independent of absolute intensity
13	Normalised contrast	$I_{norm}=\frac{x_{max}-x_{min}}{x_{max}}$	Spectral heterogeneity normalised to signal level
14	Precision	$Precision=\frac{TP}{TP+FP}$	Proportion of positive predictions that are correct
15	Recall	$Recall=\frac{TP}{TP+FN}$	Model’s ability to identify positive class instances
16	F1-score	$F1=2*\frac{\mathrm{Precision}*\mathrm{Recall}}{\mathrm{Precision}+\mathrm{Recall}}$	Harmonic mean of Precision and Recall

## Data Availability

The data supporting this study’s findings are available on request from the corresponding author.
